# Comparison of Nanostring nCounter^®^ Data on FFPE Colon Cancer Samples and Affymetrix Microarray Data on Matched Frozen Tissues

**DOI:** 10.1371/journal.pone.0153784

**Published:** 2016-05-13

**Authors:** Xi Chen, Natasha G. Deane, Keeli B. Lewis, Jiang Li, Jing Zhu, M. Kay Washington, R. Daniel Beauchamp

**Affiliations:** 1 Division of Biostatistics, Department of Public Health Sciences, University of Miami Miller School of Medicine, Miami, Florida, United States of America; 2 Department of Surgery, Vanderbilt University, Nashville, Tennessee, United States of America; 3 Affymetrix Inc., Santa Clara, California, United States of America; 4 Department of Cell and Developmental Biology, Vanderbilt University, Nashville, Tennessee, United States of America; 5 Department of Cancer Biology, Vanderbilt University, Nashville, Tennessee, United States of America; 6 Department of Pathology, Vanderbilt University, Nashville, Tennessee, United States of America; 7 Vanderbilt Ingram Cancer Center, Vanderbilt University, Nashville, Tennessee, United States of America; 8 Sylvester Comprehensive Cancer Center, University of Miami Miller School of Medicine, Miami, Florida, United States of America; Cleveland Clinic Lerner Research Institute, UNITED STATES

## Abstract

The prognosis of colorectal cancer (CRC) stage II and III patients remains a challenge due to the difficulties of finding robust biomarkers suitable for testing clinical samples. The majority of published gene signatures of CRC have been generated on fresh frozen colorectal tissues. Because collection of frozen tissue is not practical for routine surgical pathology practice, a clinical test that improves prognostic capabilities beyond standard pathological staging of colon cancer will need to be designed for formalin-fixed paraffin-embedded (FFPE) tissues. The NanoString nCounter^®^ platform is a gene expression analysis tool developed for use with FFPE-derived samples. We designed a custom nCounter^®^ codeset based on elements from multiple published fresh frozen tissue microarray-based prognostic gene signatures for colon cancer, and we used this platform to systematically compare gene expression data from FFPE with matched microarray array data from frozen tissues. Our results show moderate correlation of gene expression between two platforms and discovery of a small subset of genes as candidate biomarkers for colon cancer prognosis that are detectable and quantifiable in FFPE tissue sections.

## Introduction

The identification of colorectal cancer (CRC) patients who either more or less likely to benefit from adjuvant systemic chemotherapy after surgical resection poses a major unmet need in providing safe and effective care. The current practice may result in under-treatment of some high-risk stage II patients, and potential over-treatment of low-risk stage II and stage III patients [1]. The core obstacle is the lack of definitive diagnostic biomarkers to identify cancers with a high probability of metastasis and correspondingly poor clinical outcome that are more likely to benefit from systemic chemotherapy; and conversely, to identify those patients at very low risk (<10%) who are unlikely to derive significant benefit from chemotherapy [2–6]. Translation of microarray-based profiles into clinical diagnostics as biomarkers is complicated by the fact that the technology required to reproduce them has previously required fresh unfixed tissue samples, and there is a gap between an emerging body of genomic information and diagnostic application. The ability of gene expression signatures to predict recurrence and clinical outcome strongly argues that high- and low-risk phenotypes are molecularly encoded in primary tumors [7–9]. Nevertheless, a robust prognostic signature applicable in the clinical setting has yet to be developed for colorectal cancer due to variation in methods and procedures for preservation of surgical specimens, variation in the amount and quality of purified RNA available for analysis, tumor heterogeneity issues and the reproducibility and robustness of the assay platform. Because collection of fresh frozen tissues is not routine, a clinical test for improving staging of colon cancer will need to be designed for formalin-fixed paraffin-embedded (FFPE) tissues in order to be widely applicable.

The NanoString nCounter^®^ system has been applied to quantify gene expression of various gene signatures for multiple tissue types including FFPE samples [10–13]. We have designed a custom nCounter codeset for quantitative assessment of expression of 414 gene elements. This assay consists of multiple published gene signatures for colon cancer prognosis plus several candidate gene elements derived from ongoing studies in intestinal stem cell biology and epithelial-to-mesenchymal transition (EMT). To determine which elements of prognostic signatures can be translated from fresh frozen tissue to archival FFPE derived patient RNA samples, we systematically compared the expression results for the 414 genes from the nCounter platform using FFPE-derived tissue and from a microarray array platform using matched frozen tissues.

## Materials and Methods

### Experiment design and sample description

Human tissues used for microarray analysis were collected and annotated according to established protocols and approved by the appropriate Institutional Review Boards (IRB) at Vanderbilt University (VUMC). All tissues were collected over the time period from 1999–2011. Tumor stage was assessed by American Joint Commission on Cancer guidelines. Written informed consent was obtained from all patients prior to inclusion in the studies. Quality assessment slides were obtained to verify the diagnosis and the amount of cellular material for each sample. De-identified human tumor tissues for immunohistochemistry were obtained with VUMC IRB approval. For microarray studies, representative sections of fresh tissue specimens were flash frozen in liquid nitrogen within 20 minutes of resection and stored at −80°C until the RNA isolation step. The median (minimum-maximum) frozen tissue storage time from date of resection to RNA extraction are 455 (35–2858) days. RNA was purified from tissue sections containing >80% epithelial tumor tissue using RNeasy (QIAGEN, Valencia, CA) according to manufacturer’s instructions. Samples were hybridized to Affymetrix arrays Human Genome U133 Plus 2.0 GeneChip Expression Arrays, Santa Clara, CA), which included approximately 39,000 human genes. The samples included four healthy control patient tissues, 12 stage I, 17 stage II, 20 stage III and 15 stage IV CRC patient tissues. The detailed sample information including suvival data can be found in S1 Table.

For nCounter studies, archived tumor FFPE samples matched to frozen tissues described above were identified and processed by the Vanderbilt Translational Pathology and Imaging Core facility. Tissues were fixed in 4% paraformaldehyde, embedded in paraffin and stored at room temperature until RNA extraction. The median (minimum-maximum) FFPE tissue storage time from date of resection to RNA extraction are 1021 (364–3483) days. The first 20μm of the tissue was discarded before cutting sections for RNA extraction. Tissue sections were mounted on uncharged glass slides and one of the tissue section slides was stained with H&E for quality control from each tissue block. RNA was purified from 5μm thick tissue sections containing greater than 80% tumor using High Pure FFPE RNA Micro Kit (Roche) according to manufacturer’s instructions. A minimum of 4 sections per sample were required. The mRNA hybridization, detection and scanning were followed the protocol and performed by NanoString Technologies.

### nCounter codeset development

We designed a custom nCounter^®^ assay (NanoString Technologies, Seattle, WA) for quantitative assessment of expression of 414 gene elements. This multiplexed assay can detect expression of up to 800 transcripts at very low mRNA concentrations (0.1fM/1 copy per cell) [14] even in RNA samples that are significantly degraded, as long as more than 20% of the sample has RNA fragments of greater than 300 base pairs [13]. Table 1 lists all gene signatures involved in the nCounter Codeset. We included gene elements from our 34-gene signature [9], as well as elements from published signatures [7, 8, 15–24] in the nCounter assay, selecting those that were represented in ≥2 signatures. Fifty additional candidate genes of interest related to EMT and the study of metastatic behavior in cancer cells (eg, E-cadherin, Smad4, Zeb1, Snail, Slug, Twist, beta-catenin, etc.) were also added to the assay. The full list with gene symbols and the sources of the 414-genes can be found in S2 and S3 Tables.

**Table 1:** Sources of Nanostring codeset of 414-gene list.

Source	Genes included	Description
Eschrich 2005 [8]	21	Outcomes-based supervised analysis of 78 cases, stages I-IV
Barrier 2006 [7]	18	Outcomes-based supervised analysis of 50 cases, stage II only
Ki 2007 [19]	36	Differential expression between 25 primary colon tumors with and without synchronous metastasis
Lin 2007 [20]	24	Outcomes-based supervised analysis of 2 cohorts (n = 149, all stages; n = 55, stage I & II)
Wang 2004 [24]	23	Outcomes-based supervised analysis of 74 Duke’s B cases.
Grade 2007 [17]	121	Differential expression of 73 stage II & stage III cases based lymph node involvement
Paik 2004 [21]	9	Clinically approved classifier for recurrence in tamoxifen treated, node-negative breast cancer
Kanies 2008 [18]	5	Experimentally derived signature of Epithelial-to-Mesenchymal Transition
Fritzmann 2009 [15]	82	Differential expression of 95 stage matched primary tumors with and without synchronous metastasis
Smith 2010 [9]	34	Experimentally derived classifier based on immunocompetent mouse model of metastasis
Salzar 2011 (Coloprint) [22]	13	Supervised analysis of 188 cases based on metastasis-free survival
Ga**r**man **2008** [16]	50	Outcomes-based supervised analysis of 52 stages I and stage II cases.
Tripathi 2014 [23]	57	Systems-based classifier based on experimental mouse model of metastasis

### Bioinformatics and statistical data analysis

Affymetrix microarray data were normalized using the Robust MultiChip Averaging (RMA) algorithm as implemented in the Bioconductor package *Affy* [25]. The batch effects were adjusted by ComBat approach available in the Bioconductor package *SVA* [26]. NanoString nCounter data quality control and normalization were performed using R package *NanoStringNorm* [27]. For group comparisons, t-test in the *Limma* package in Bioconductor was used to identify differentially expressed probe sets between the tumor and normal tissues in both Affymetrix and nCounter platforms [28]. For survival analysis, the Cox proportional hazard was applied to test the association with overall survival outcomes for each candidate gene using R package *survival*.

In this study, in order to make direct comparison between Affymetrix HGU133plus 2 and nCounter gene expression data, we first found the best-matched Affymetrix microarray probes to NanoString nCounter probes. Toward this end, we used gene symbol or transcript ID to link NanoString nCounter IDs and Affymetrix Probe IDs. If the gene had only one unique probe, then we used that Probe ID as the match for NanoString. Otherwise, if the gene had multiple probes, then we picked the one with the largest Smith–Waterman alignment score, which was calculated by Smith-Waterman algorithm to identify homologous regions between sequences by searching for optimal local alignments, between NanoString nCounter and Affymetrix Probe sequences as the match for nCounter [29]. Both microarray and nCounter data are available in Gene Expression omnibus (GEO) database (GSE62932).

## Results

### Quality of extracted RNA

RNA Integrity Numbers (RIN) values were obtained using an Agilent Bioanalyzer instrument for both frozen and FFPE derived RNA samples. The instrument software generates a RIN score based on its entire electropherogram. RIN values range from 1–10, with 1 being totally degraded RNA and 10 being high quality (intact) RNA. For microarray studies, a cut-off of RIN = 7.0 was used. RIN values ranged from 7–10, with a median of 8.5 and mean of 8.4. For nCounter studies, RIN values ranged from 0–8, with a median of 2.4 and mean of 2.5. In addition, to be acceptable for analysis for nCounter, ≥20% of the RNA sample had to contain fragments of at least 300 base pairs in length. The details of the results were included in S4 Table.

### Data quality of nCounter and microarray

**Fig 1:**
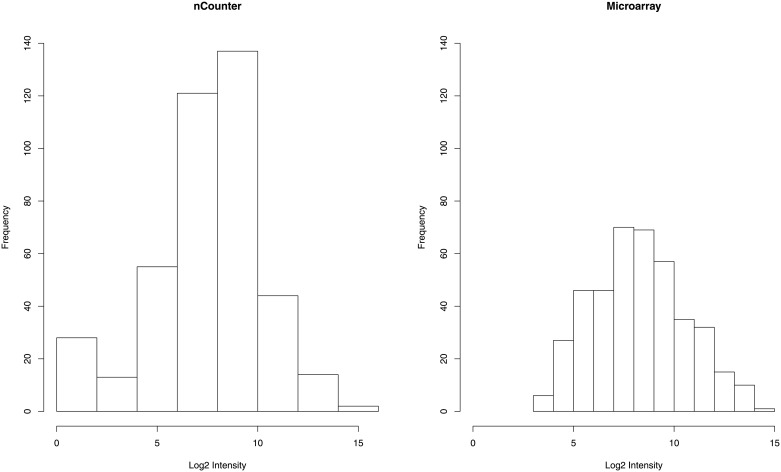
Histograms for nCounter and microarray. The frequency of gene probes (414 elements total) is graphed against median normalized expression values across the x-axis.

After pre-processing and normalization, log2 transformed data from 414 genes of both microarray and nCounter were compared. The distributions of signal intensities for all samples are displayed with boxplots in S1 Fig. To draw the gene expression distribution, each of the 414 genes was represented by the median value for 68 samples. Fig 1 compares gene expression distributions based on nCounter and microarrays. nCounter data showed a bi-modal distribution that is likely due to the RNA degradation in FFPE samples. In more detail, we found that ~70% of genes had median normalized expression values in the 5–10 arbitrary unit range on both platforms. In contrast, about 15% of the genes showed very low normalized expression values (≤ 5 arbitrary units) on the nCounter platform while only 8% of the genes performed as poorly on the microarray platform. Conversely, only 14% of the genes showed expression robust median expression values (> 10 arbitrary units) on the nCounter platform while closer to 23% of the genes gave robust signals on the microarray platform. In summary, the amount of useable data achieved using the nCounter platform was low relative to that achieved on the microarray platform.

To examine the reproducibility of the nCounter platform, six FFPE samples were selected to generate two more technical replicates for each sample. S2 Fig shows the scatterplots of the log2-transformed counts between all pairs of replicates. The average correlation coefficient is 0.983, which indicates that the nCounter platform is reproducible.

### Sample and gene correlations between nCounter and microarray

**Fig 2:**
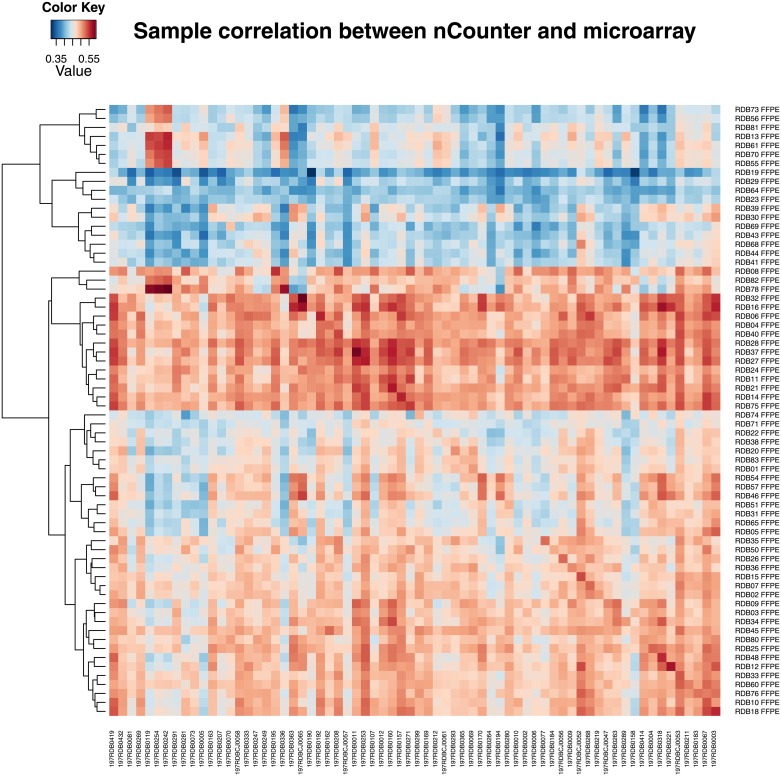
The heatmap of pairwise correlations between individual matched FFPE and fresh frozen samples. X-axis represents the fresh frozen samples and y-axis represents matched FFPE samples.

For each pair of FFPE sample quantified by nCounter and fresh frozen sample quantified by microarray, the Pearson correlation was calculated based on normalized, log2 transformed gene expression values of the 414 genes. The mean correlation was 0.501 with 95% bootstrap confidence interval (0.412, 0.589), and the minimum and maximum correlation were 0.337 and 0.591 respectively. The heatmap in Fig 2 displays all pairwise correlations between FFPE and fresh frozen samples. The analysis based on Spearman correlation showed similar results, which suggested the correlation pattern between samples was not affected by different normalization procedures since the normalization methods don’t change the gene rankings within each sample.

**Fig 3:**
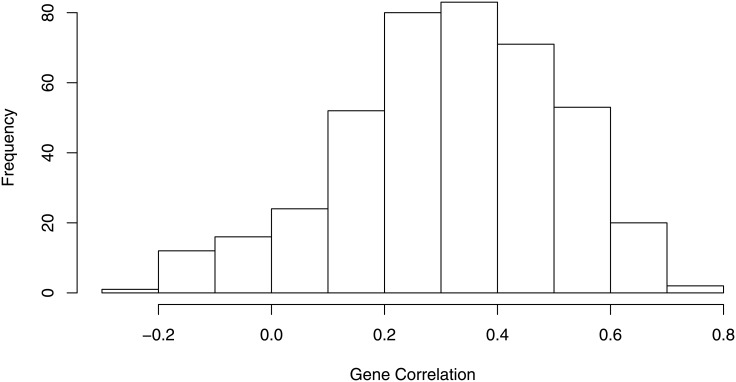
The histogram of gene-wise correlations between FFPE and fresh frozen samples. X-axis represents Pearson correlation of each of 414 genes between fresh frozen samples and FFPE samples and y-axis represents the frequency.

For each of 414 genes, the Pearson correlation was also calculated between two tissue types. We plotted the histogram of gene-wise correlation coefficients in Fig 3. The mean correlation of the 414 genes was 0.315 with 95% bootstrap confidence interval (0.248, 0.385), and the minimum and maximum correlation were -0.250 and 0.784 respectively. Gene-wise correlation represents the concordance of gene expression values from two expression platforms between two matched tissue type. Due to nCounter’s single probe design, some genes did not match well between two platforms, which may have contributed to the weak or negative correlations. It is reasonable to observe this moderate level of concordance because the variations in gene expressions were from two different platforms and tissue types. The medium level of concordance between genes also led to the ambiguous pattern between fresh frozen and FFPE samples displayed in Fig 2.

### Comparison of gene expression between nCounter and microarray

**Fig 4:**
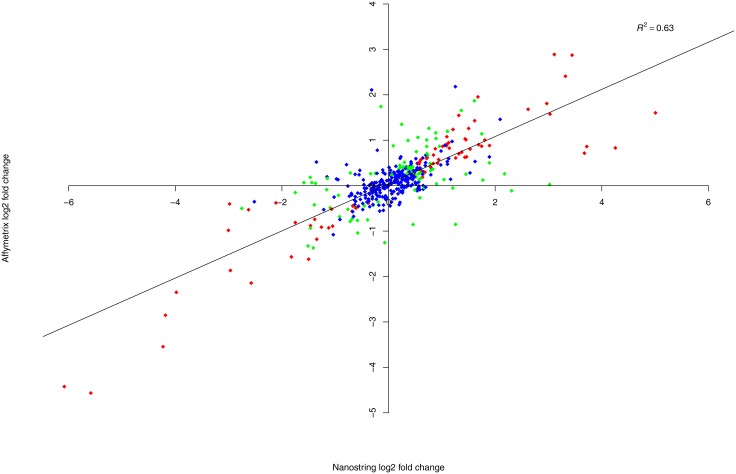
Scatterplot of log2 fold change. Log2 fold change in tumor to normal signal intensity for 414 genes comparing results from nCounter (x-axis) and microarray (y-axis). The red, green and blue dots represent 76, 90 and 248 genes significantly differentially expressed in both, either, and neither platforms respectively.

After preprocessing and normalization, to detect differentially expressed genes between 64 tumor and 4 normal samples in nCounter or microarray platforms separately, we used regularized t-test as implemented in the *Bioconductor* package *LIMMA*. Using nominal p-value 0.05 as the cutoff, 76 genes were identified as significantly differentially expressed genes in both platforms. Among the 76 genes, 40 and 26 genes were overexpressed and downregulated in cancer samples. There were 30 and 60 significant genes discovered in only one platform by nCounter and microarray respectively, and 248 genes were not significant by either platform. The complete gene testing lists including coefficient, nominal p-value and false discovery rate can be found in S5 and S6 Tables for nCounter and microarray, respectively. Fig 4 shows the comparison of log2 fold change in tumor to normal signal intensity between nCounter and microarray for each of 414 genes that were selected for the nCounter codeset. The red, green and blue dots represent 76, 90 and 248 genes significantly differentially expressed in both, either, and neither platforms respectively. If we use FDR 0.1 as the cutoff, there were 48, 72 and 294 genes significantly differentially expressed in both, either, and neither platforms respectively.

### Association of nCounter and microarray gene expression with survival outcome

**Fig 5:**
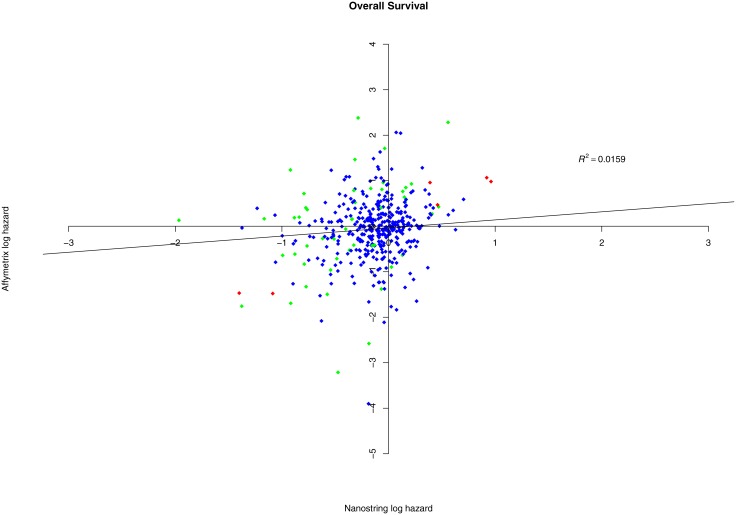
Scatterplot of log hazard ratio. Log hazard ratio for tumor to normal signal intensity for 414 genes vs. overall survival outcomes comparing results from nCounter (x-axis) and microarray (y-axis). The red, green and blue dots represent 6, 49 and 359 genes significantly associated with survival outcomes in both, either, and neither platforms respectively.

We also examined the association of gene expression with overall survival outcomes of 64 patients using a Cox proportional hazard model. Using a nominal p-value 0.05 as the threshold, there were 6 significantly associated genes including PPL, CCND1, EXT2, S100A11, USP9X, and PHLDA1 by both platforms, 25 significant genes by nCounter, and 24 significant genes by microarray. PPL, CCND1, S100A11, and PHLDA1 were associated with higher hazard, and EXT2 and USP9X were associated with better prognosis. The majority of the genes (359 genes) were not significantly associated with overall survival outcomes by either platform due to relatively small sample size. The complete gene testing lists including coefficient, nominal p-value and false discovery rate or this survival data analysis are in S7 and S8 Tables for nCounter and microarray, respectively. Fig 5 shows the log hazard ratios for overall survival outcomes of the 414 genes compared between nCounter and microarray data, with the red, green and blue dots indicating the 6, 49 and 359 genes having significant survival association in the two, one and neither platforms. For the six genes identified by both platforms, previous studies showed that CCND1 and S100A1can be used as biomarkers for colon cancer prognosis [30, 31], and the inhibition of USP9X might increase the tumor cell sensitivity to some chemotherapeutic agents [32, 33].

## Discussion

The quality of mRNA transcript quantification in FFPE samples is crucial for translational cancer research. In this study, our comparisons were based on the measurements of NanoString’s nCounter platform using FFPE-derived samples and Affymetrix’s Human Genome U133 Plus 2.0 GeneChip Expression Arrays on patient matched fresh frozen tissue. Ideally, mRNA quantification using the same platform on matched FFPE and frozen tissues would make a more direct comparison. However, in order to determine whether gene signatures developed using fresh frozen tissues are applicable for clinical samples, comparisons between the two platforms using matched, but differentially archived tissues, is required.

The nCounter-based mRNA profiling using FFPE technical replicates showed high reproducibility of the platform similar to the previous study [10]. However, the average correlation coefficients were 0.501 and 0.315 for paired FFPE/fresh-frozen sample correlations and matched gene correlations respectively. These results indicated moderate correlations between nCounter (FFPE) and microarray (fresh-frozen) data.

For tumor and normal group comparisons, 166 of the 414 genes were identified as significantly differentially expressed genes by either platform and that of these 166 genes, 76 genes (46%) were expressed concordantly between the two platforms. Demonstrating an association with survival outcomes is more challenging, only 11% genes (6 genes) among significantly associated genes (55 genes) by either platform were concordant.

Multiple factors can affect these experimental results. The nCounter platform, by its very nature, represents a custom build of candidate gene elements as a biomarker, thus the selection of genes in an nCounter codeset inherently limits *in silico* comparisons with other published studies. The degree of RNA degradation is another challenging issue facing transcriptome profiling strategies using FFPE-derived samples. For example, a recent study on hepatocellular carcinoma demonstrated that mRNA transcript measurements achieved using the nCounter system for archived sectioned FFPE samples compared poorly with freshly cut FFPE tissues [11]. This issue relates both to the problem of variation in sample preparation and to the issue of inherent tumor heterogeneity reflected in measurable molecular differences between adjacent tissue sections. In this study, NanoString Technologies provided the single probe design based upon transcript frequency for our gene panel, but using the multiple probe design might be a strategy to improve the nCounter data quality.

Archived FFPE preserved tumor tissue remains the most available tissue specimen source for large-scale development and validation of prognostic and predictive gene signatures for colon cancer. Identification of subgroups of archival tumor tissues producing higher quality RNA samples for expression profiling is one approach to improving biomarker development from these samples. Improvements in technology such as new FFPE RNA extraction protocols, new FFPE RNA amplification and labeling methods, and modified data processing workflows can also help to side-step issues of RNA degradation and enhance the transcriptome data quality from RNA-Seq or microarray [34–36]. In the current study, we showed that the gene signature developed on microarray gene expression data using fresh frozen samples may not be directly applicable to nCounter data based on FFPE samples. However, we demonstrated that a small subset of genes had stable expression patterns in both the nCounter and microarray platforms, and independent of whether the lysates were derived from fresh frozen or FFPE-derived samples. Follow-up studies on these genes may help lead to more clinically useful biomarkers for colon cancer.

## Supporting Information

S1 Fig The distributions of signal intensities of 68 samples (individual box plots) by nCounter and microarray. (PDF)

S2 Fig Scatterplot of the normalized counts from 414 individual gene elements using a split sample from six FFPE preserved tumor extractions (blinded technical replicates) on the nCounter platform. (PDF)

S1 Table The clinical information of 68 samples. (CSV)

S2 Table The full list with gene symbols and the sources of the 414-genes for nCounter codeset. (CSV)

S3 Table The list of matched Affymetrix probe IDs of the 414-genes for nCounter codeset. (CSV)

S4 Table The RNA extraction RIN values (CSV)

S5 Table The differentially expressed gene testing results for nCounter. (CSV)

S6 Table The differentially expressed gene testing results for microarray. (CSV)

S7 Table The testing results of association with survival outcomes for nCounter. (CSV)

S8 Table The testing results of association with survival outcomes for microarray. (CSV)
