# Supplementary figures and images for: Comparison of Nanostring nCounter® Data on FFPE Colon Cancer Samples and Affymetrix Microarray Data on Matched Frozen Tissues
Source: PLoS One. 2016 May 13;11(5):e0153784. doi: 10.1371/journal.pone.0153784 (PMC4866771; doi:10.1371/journal.pone.0153784)

nCounter

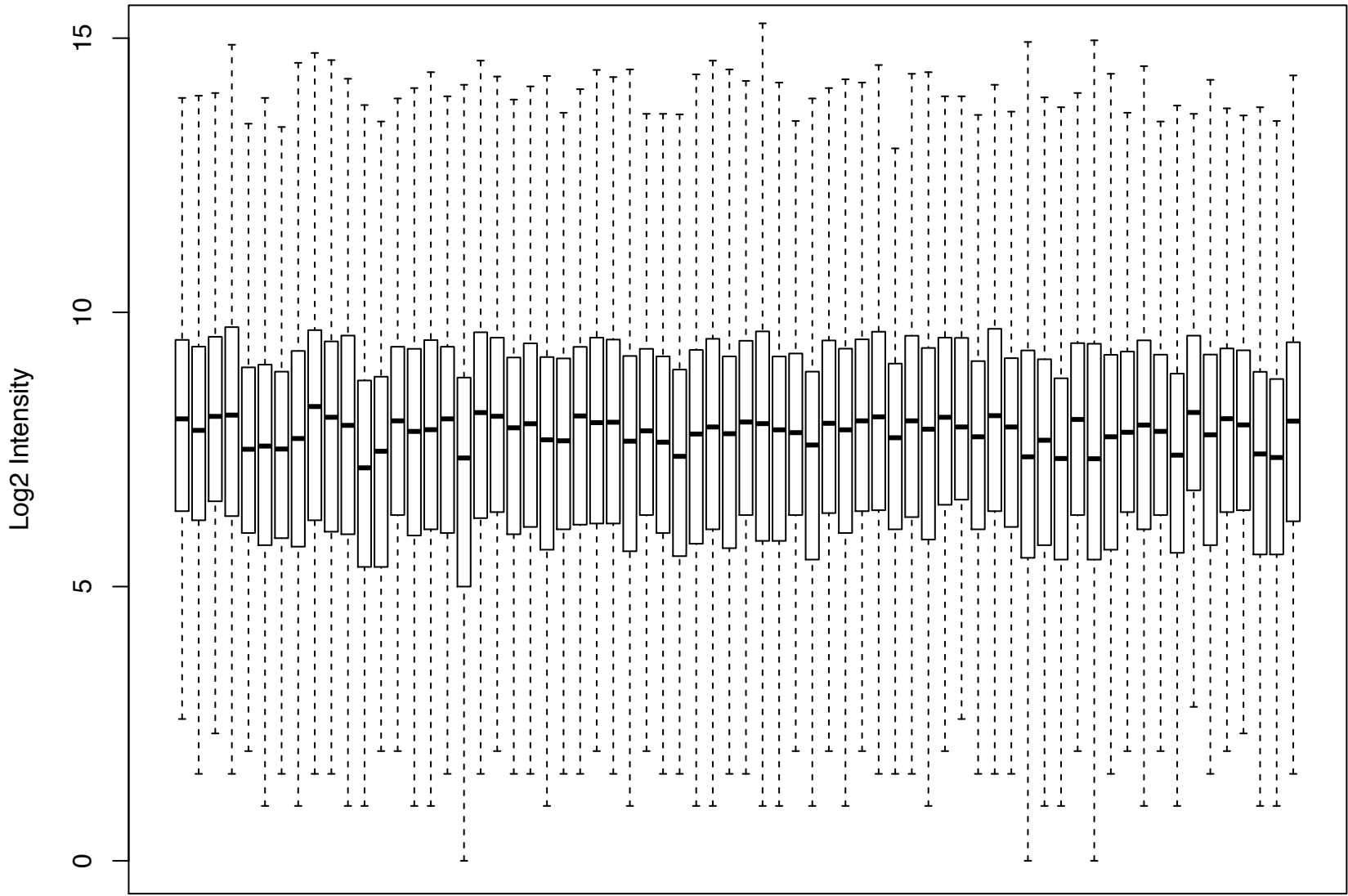

nCounter sample

Microarray

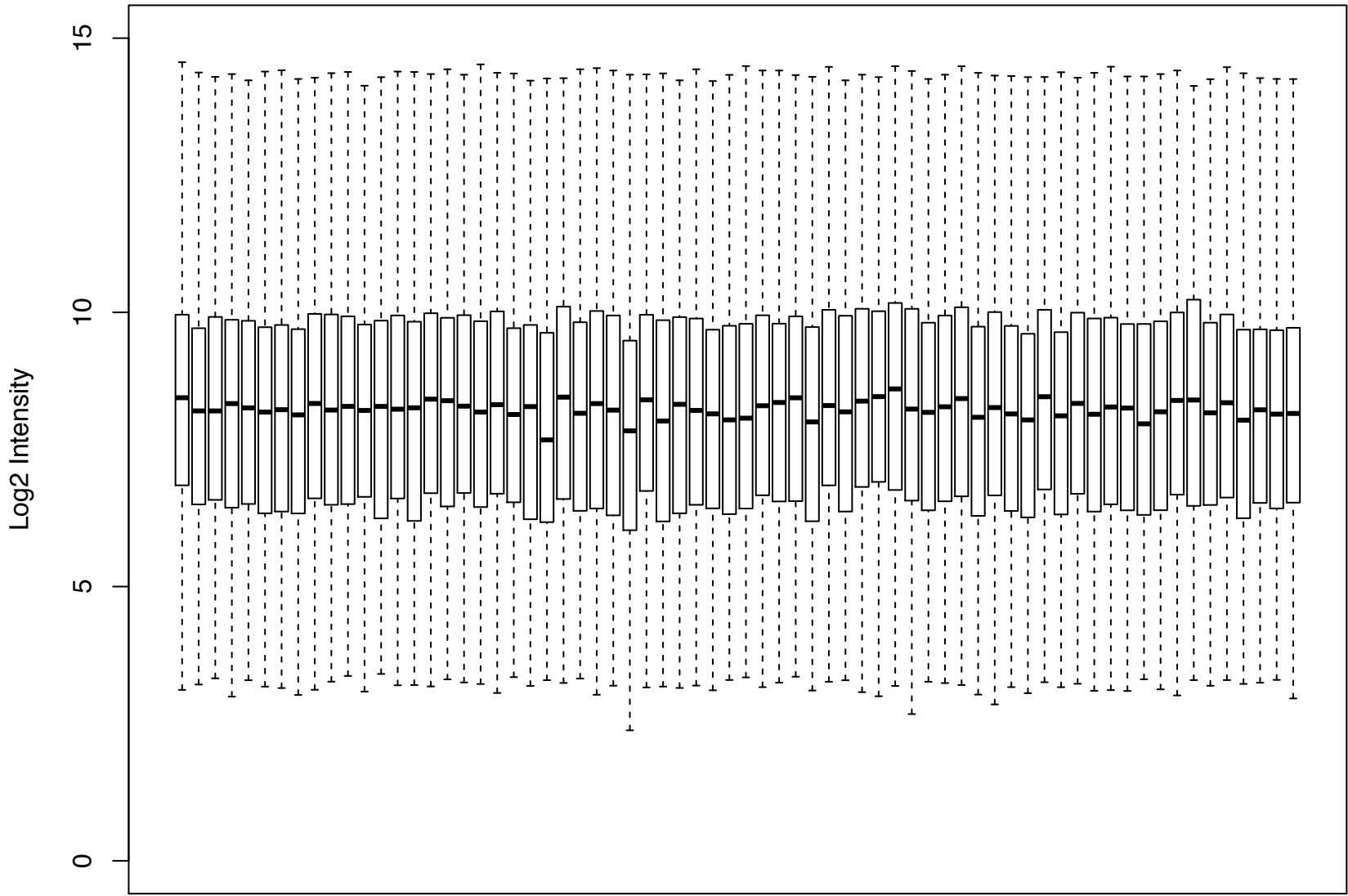

microarray sample

Supplement: S1 Fig — (PDF) [file pone.0153784.s001.pdf]
