# Supplementary material for: Comparison of Nanostring nCounter® Data on FFPE Colon Cancer Samples and Affymetrix Microarray Data on Matched Frozen Tissues
Source: PLoS One. 2016 May 13;11(5):e0153784. doi: 10.1371/journal.pone.0153784 (PMC4866771; doi:10.1371/journal.pone.0153784)
Supplement: S2 Fig — (PDF) [file pone.0153784.s002.pdf]

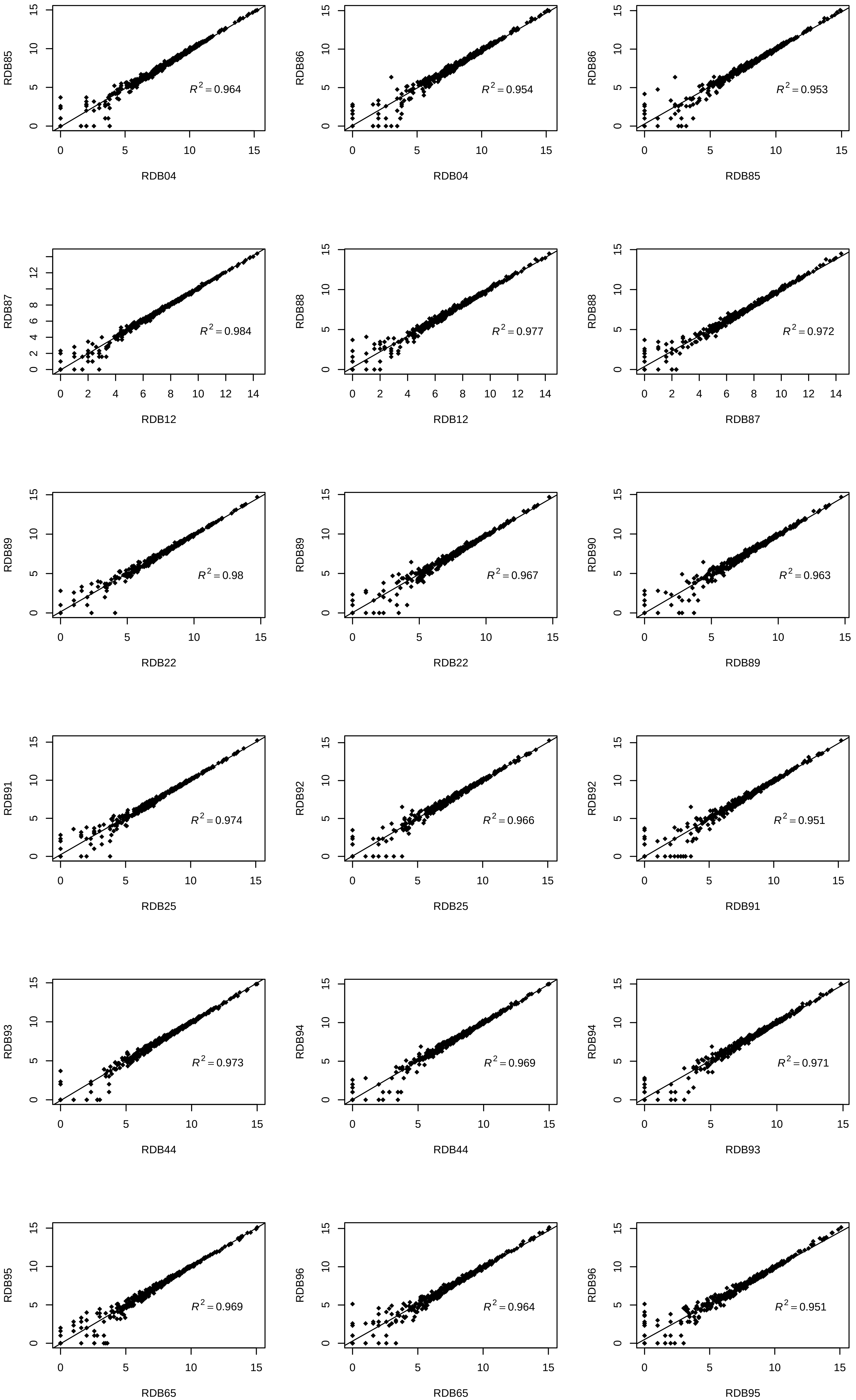

RDB85
RDB04
$R^2 = 0.964$
RDB86
RDB04
$R^2 = 0.954$
RDB86
RDB85
$R^2 = 0.953$
RDB87
RDB12
$R^2 = 0.984$
RDB88
RDB12
$R^2 = 0.977$
RDB88
RDB87
$R^2 = 0.972$
RDB89
RDB22
$R^2 = 0.98$
RDB89
RDB22
$R^2 = 0.967$
RDB90
RDB89
$R^2 = 0.963$
RDB91
RDB25
$R^2 = 0.974$
RDB92
RDB25
$R^2 = 0.966$
RDB92
RDB91
$R^2 = 0.951$
RDB93
RDB44
$R^2 = 0.973$
RDB94
RDB44
$R^2 = 0.969$
RDB94
RDB93
$R^2 = 0.971$
RDB95
RDB65
$R^2 = 0.969$
RDB96
RDB65
$R^2 = 0.964$
RDB96
RDB95
$R^2 = 0.951$
